# Evaluation of Hematological Profile in Oral Submucous Fibrosis

**DOI:** 10.7759/cureus.21926

**Published:** 2022-02-05

**Authors:** Mohammed Abidullah, Kavitha Gaddikeri, Bushra Anjum, Swapneel Vairagare, Kulkarni Tarani, Swetcha Seethamsetty

**Affiliations:** 1 Department of Dental and Biomedical Sciences, Al Baha University, Al Baha, SAU; 2 Department of Oral Pathology, Employees State Insurance Corporation (ESIC) Dental College, Gulburga, IND; 3 Department of Oral and Maxillofacial Pathology, Panineeya Institute of Dental Sciences and Research Centre, Hyderabad, IND; 4 Department of Conservative Dentistry and Endodontics, Nanded Rural Dental College and Research Center, Vishnupuri, IND; 5 Department of Periodontics, Sharad Pawar Dental College, Wardha, IND; 6 Department of Oral and Maxillofacial Surgery, Panineeya Mahavidyalaya Institute of Dental Sciences, Hyderabad, IND

**Keywords:** vitamin b12, oral submucous fibrosis, leukoplakia, iron status, hemtological profile

## Abstract

Background: The aim of the study is to assess the hematological profile in oral submucous fibrosis (OSMF) patients.

Methods: The study's participants (100) were divided into two groups. Group I consisted of fifty areca nut chewers with complaints of burning sensations, blanching, and stiffness of the oral mucosa. Group II consisted of fifty healthy patients as controls. A hematological profile was estimated in all subjects.

Results: The control group had a mean hemoglobin (Hb) of 13.87±1.26 g/dL, while the OSMF group had a mean Hb of 11.03±2.16 g/dL (P=0.001). The mean serum iron level in the control group was 120.36±41.22 g/dL, while it was 44.97±13.45 g/dL in the OSMF group (P=0.001). The control group's mean serum vitamin B12 values were 424.77±110.95 g/dL, while the OSMF group's was 210.11±44.88 g/dL (P=0.001). In the research population, 47 patients had iron deficiency. The odds ratio (OR) was 28.11, which meant that the high prevalence of iron deficiency was predicted 28.11 times more often than in the control group.

Conclusion: As part of a biochemical assessment, iron status is assessed as part of a prevention mechanism for people who are at high risk. Biochemical testing has been suggested as a potential tool for mass screening OSMF patients.

## Introduction

Oral cancer is often preceded by clinically noticeable lesions that are initially noncancerous, termed as precancerous lesions like oral submucous fibrosis (OSMF) [1,2]. Molecular markers found in body fluids such as urine, blood, and saliva can help diagnose oral cancer, determine prognosis, and monitor its development [3,4]. During tumor growth, various substances in the serum change quantitatively; these are known as tumor markers, which appear well before frank cancer has arisen. We can determine whether a single person with the underlying biochemical defect will develop cancer or not at a later date even in oral potentially malignant disorders [5-7]. Low levels of hemoglobin (Hb) can impair the consistency of the oral mucosa [8]. Hemoglobin levels, especially serum iron levels, are used to determine nutritional status as biochemical markers [9]. Iron, vitamin B-12, and folate deficiency will compromise the integrity of the oral mucosa. OSMF has been linked to hematological anomalies, including an elevated erythrocyte sedimentation rate (ESR), reduced serum iron, and increased iron-binding ability [10].

## Materials and methods

Our study was carried out between April 2018 and October 2018, after receiving the approval of the institutional ethics committee with vide no. "SBPDCH/BDR/2019/EST/630." After obtaining informed consent, the patient or family, if the patient was not in good health, were asked to have a full medical history. Patients were informed about the procedure's technique, costs, advantages, outcomes, and related complications.

Two population mean formulae were used to calculate the sample size using G-Power, version 3.1 (HHU, Dusseldorf, Germany), by considering the following assumptions: 95% confidence interval (two-sided), 80% power, and the ratio of cases to control group was 1:1. Using the mean and standard deviation (SD) of hemoglobin calculation from the previous study, 4.63 and 0.59 for symptomatic OMSF patients and 4.83 and 0.72 for the control group, we got a total sample size of 55 for each group. Taking consideration of 10% dropouts, we decided to take 50 patients in each group. The patients were randomized into two groups using the chit and box method.

The sample population was classified into two categories. Group I consists of 50 patients above 18 years with an areca nut habit and clinical complaints of burning symptoms and blanching of the oral mucosa, and a clinical diagnosis of OSMF, according to Arakeri et al. [11]. Group II consists of 50 healthy patients (as control). The patients with stage IV OSMF patients or previously treated cases with OSMF were excluded from the study. 

Estimation of iron, hemoglobin, and red cell indices

Five milliliters of fasting venous blood was collected and Sahli's protocol was used for hemoglobin calculation and the Ferrone system for estimating serum iron levels. The samples were analyzed for trace elements (copper, iron) by means of atomic absorption spectrometry and a differential pulse anodic stripping voltmeter (DPASV) [12]. The chemiluminescent microparticle intrinsic factor assay for the quantitative assessment of vitamin B12 in human serum was used [13].

Statistical analysis

For the statistical method, IBM SPSS Statistics for Windows, version 20 (IBM Corp., Armonk, NY, USA) is used. The Chi-square test was used to measure the mean values and standard deviations for all of the groups. The Kolmogorov-Smirnov test was used to determine the normality of different parameters in the control and sample classes. The independent t-test was used to analyze more than two means at the same time, i.e., whether there was a substantial difference in serum iron, vitamin B12, and Hb between the two classes. Karl Pearson's correlation coefficient approach was used to analyze correlations between different parameters in the control and sample groups. A logistic regression analysis was used to determine which hematological component was more important for OSMF. The significance was set at P≤0.05.

## Results

Table 1 shows the gender distribution in the OSMF and control groups. When the two demographic classes were matched for age, the majority of cases between 30 and 40 years old seemed to be statistically significant (p = 0.0123; control and study).

**Table 1:** Demographic profile of the patients in control and study groups

	Control group		Study group	
Gender	Number of patients = 50	Percentage	Number of patients = 50	Percentage
Male	29	58	23	46
Female	21	42	27	54
Age in years				
Below 30	8	16	5	10
30–40	22	44	24	48
40–50	14	28	12	24
Above 50	6	12	9	18
Mean age	32.47 ± 11.71 years.		40.11 ± 11.37 years	

The majority of patients had grade II and III OSMF (Table 2). Table 3 displays the results of an experimental t-test comparing the mean values of hematological parameters in the sample and control classes, and the results were statistically significant (p=0.0001). The control group had a mean Hb of 13.87±1.26 g/dL, while the OSMF group had a mean Hb of 11.03±2.16 g/dL (p=0.001). The mean serum iron level in the control group was 120.36±41.22 g/dL, while it was 44.97±13.45 g/dL in the OSMF group (p=0.001). The control group's mean serum vitamin B12 values were 424.77±110.95 g/dL, while the OSMF groups' were 210.11±44.88 g/dL (p=0.001).

**Table 2:** Patient distribution in various grades of oral submucous fibrosis

Various grades of oral submucous fibrosis	Number of patients	Percentage
I	10	20
II	20	40
III	20	40

**Table 3:** Comparison of control and study groups by independent t-test

Parameters	Mean ± SD		T-value	P-value
	Control group	Study group		
Hb (g/dL)	13.87±1.26	11.03±2.16	6.7811	<0.001
PCV	43.12±5.42	34.17±6.37	5.6122	<0.001
MCV (fl)	87.12±6.53	69.87±9.23	8.0812	<0.001
MCH	28.21±2.77	24.96±4.31	3.6487	<0.001
MCHC	31.97±1.04	30.04±2.52	2.9569	<0.01
Iron (mg/dL)	120.36±41.22	44.97±13.45	10.4063	<0.001
Vitamin B12 (pg/Ml)	424.77±110.95	210.11±44.88	10.6241	<0.001

The values of Hb, packed cell volume, mean corpuscular volume, mean corpuscular Hb (MCH), and MCHC levels in the OSMF stage III had a strong association with serum iron levels (Pearson correlation values: 0.6970, 0.6330, 0.4503, and 0.2210, respectively). The study group had 47 patients with iron deficiency, while the control group had only seven. The odds ratio (OR) was 28.11, which meant that the high occurrence of iron deficiency was predicted 28.11 times more often in the study group than in the control group. Vitamin B12 deficiency was found in 28 patients in the study group versus two patients in the control group, with the study group showing a higher rate of deficiency than the control group. The forty OSMF patients had a mean mouth opening of 13.9. Every patient had OSMF involvement in the soft palate, retromolar region, and buccal mucosa, with labial mucosa involvement in 32 patients (64%), the floor of the mouth involvement in 25 patients (50%), and tongue involvement in 20 patients (40%). 18 of the 50 OSMF patients had three-site involvement, 20 had four-site involvement, and eight had no involvement at all (Table 4).

**Table 4:** Logistic regression analysis of the study group by hematological parameter *P<0.05. SD: standard deviation, CI: confidence interval, OR: odds ratio

Parameters	Control group	Study group	OR	95% CI for OR		P-value
				Lower	Upper	
Hb (g/dL)						
Deficiency	9	43	0.92	0.11	6.23	0.9741
Normal	41	7				
PCV						
Deficiency	2	23	2.71	0.16	50.96	0.5440
Normal	48	27				
MCV (fl)						
Deficiency	5	43	3.12	0.22	43.77	0.4317
Normal	45	7				
MCH						
Deficiency	12	32	0.22	0.03	1.93	0.1630
Normal	38	18				
MCHC						
Deficiency	9	28	0.24	0.02	2.74	0.2598
Normal	41	22				
Iron (mg/dL)						
Deficiency	7	47	28.11	1.12	687.74	0.0463*
Normal	43	3				
Vitamin B12 (pg/Ml)						
Deficiency	2	28	3.87	0.42	43.36	0.2871
Normal	48	22				

## Discussion

The classification by Arakeri et al. [11] was used because it aids in effectively classifying information, collecting details, proper contact, prognosis, and making disease features easier to understand, which may be readily implemented by trainees and clinicians. It is a three-tiered structure that distinguishes between medical, surgical, and malignant disease treatment. In this study, OSMF patients had reduced serum iron, hemoglobin, and vitamin B12 than controls. Other experiments performed around the world yielded similar findings [12-14].

Wang et al. [15] found that folic acid and vitamin B12 levels were shown to be lower in OSMF patients. Another study conducted by Wahi et al. [14] discovered that the OSMF community had lower levels of vitamin B12 and vitamin C. In comparison to these two findings, the current research looked at the prevalence of vitamin B12 and hematological parameters. Most experiments did not look at the RCIs.

Iron is a critical component of nucleic acids and collagen, as well as being involved in the formation and preservation of the oral mucosa [13]. Iron deficiency anemia (IDA) is characterized by weakness, achlorhydria, epithelial atrophy, lack of concentration, irritability, dyspnea, and impaired memory due to low serum iron levels. Dysphagia is due to the presence of irregular esophageal webs that are predisposed to becoming malignant. Epithelial atrophy, thick corium, and enhanced collagen synthesis are the signature histopathological characteristics of OSMF [16-19].

IDA was higher in 47 OSMF patients (94%) than in stable controls. Many experiments have come up with contradictory findings. Wahi et al. [14] found anemia in 6% of males and 11% of female OSMF patients, but the prevalence of anemia in subjects did not vary significantly from the controls.

Bhardwaj et al. [12] found a gradual reduction in serum iron and Hb levels from stage I OSMF to stage IV OSMF in 120 participants, as was found in the current study. The findings of our analysis are similar to Karthik et al. [20], Khanna et al. [21], and Lavina et al. [22].

OSMF causes iron deficiency as a result of inadequate eating patterns and burning sensations, making it impossible to consume a normal diet and resulting in poor feeding. Studies described cases in which IDA caused the growth of OSMF, which was treated with iron supplements and antioxidants given orally [12-14]. 

In advanced circumstances, the deficiency may be aggravated by the effect of a poor diet due to poor food consumption. They also discovered that vitamin and iron deficiency, and the host's malnutrition, cause a disruption in the lamina propria's inflammatory reparative reaction, resulting in poor healing and scarification, and eventually, OSMF. Tobacco use leads to a deficiency of micronutrients. Low vitamin B12 levels may not be the primary cause of cancer, but they may work in concert with carcinogens, genetics, and environmental factors to speed up the malignant transformation process. Because of its function in DNA synthesis and repair, it plays an important role in cancer prevention. While the defects may not be carcinogenic, they may increase the sensitivity of other carcinogens' behavior [2-15]. As a result, many issues exist, necessitating more in-depth analysis. Thus, plenty of questions still remain, which necessitates more detailed research to recognize the connection between OSMF and the hematological picture.

## Conclusions

Our study revealed that OSMF has a high malignant transformation rate and also that low serum iron levels are associated with head-and-neck cancers. Hence, it is vital to identify and treat the low serum iron and vitamin B12 levels in OSMF patients. It is also advised that these individuals be given vitamin and iron supplements. Future studies must be targeted towards the systemic usage of iron and vitamins in OSMF patients so as to halt its progression to oral cancer, thus preventing malignant transformation. Biochemical research may be useful in the mass screening of OSMF. Further studies are required in this area in order to establish the precise part that these parameters play in OSMF pathogenesis.
